# Low Global Diversity of *Candidatus* Microthrix, a Troublesome Filamentous Organism in Full-Scale WWTPs

**DOI:** 10.3389/fmicb.2021.690251

**Published:** 2021-06-25

**Authors:** Marta Nierychlo, Caitlin M. Singleton, Francesca Petriglieri, Lisette Thomsen, Jette F. Petersen, Miriam Peces, Zivile Kondrotaite, Morten S. Dueholm, Per H. Nielsen

**Affiliations:** Center for Microbial Communities, Department of Chemistry and Bioscience, Aalborg University, Aalborg, Denmark

**Keywords:** activated sludge, *Ca.* Microthrix, bulking, diversity, genome, Raman, fish, amplicon sequencing

## Abstract

*Candidatus* Microthrix is one of the most common bulking filamentous microorganisms found in activated sludge wastewater treatment plants (WWTPs) across the globe. One species, *Ca.* M. parvicella, is frequently observed, but global genus diversity, as well as important aspects of its ecology and physiology, are still unknown. Here, we use the MiDAS ecosystem-specific 16S rRNA gene database in combination with amplicon sequencing of Danish and global WWTPs to investigate *Ca.* Microthrix spp. diversity, distribution, and factors affecting their global presence. Only two species were abundant across the world confirming low diversity of the genus: the dominant *Ca.* M. parvicella and an unknown species typically present along with *Ca.* M. parvicella, although usually in lower abundances. Both species were mostly found in Europe at low-to-moderate temperatures and their growth was favored in municipal WWTPs with advanced process designs. As no isolate is available for the novel species, we propose the name “*Candidatus* Microthrix subdominans.” Ten high-quality metagenome-assembled genomes recovered from Danish WWTPs, including 6 representing the novel *Ca.* M. subdominans, demonstrated high genetic similarity between the two species with a likely preference for lipids, a putative capability to reduce nitrate and nitrite, and the potential to store lipids and poly-P. *Ca.* M. subdominans had a potentially more versatile metabolism including additional sugar transporters, higher oxygen tolerance, and the potential to use carbon monoxide as energy source. Newly designed fluorescence *in situ* hybridization probes revealed similar filamentous morphology for both species. Raman microspectroscopy was used to quantify the *in situ* levels of intracellular poly-P. Despite the observed similarities in their physiology (both by genomes and *in situ*), the two species showed different seasonal dynamics in Danish WWTPs through a 13-years survey, possibly indicating occupation of slightly different niches. The genomic information provides the basis for future research into *in situ* gene expression and regulation, while the new FISH probes provide a useful tool for further characterization *in situ*. This study is an important step toward understanding the ecology of *Ca.* Microthrix in WWTPs, which may eventually lead to optimization of control strategies for its growth in this ecosystem.

## Introduction

The activated sludge process is the most common wastewater treatment technology applied worldwide. In recent years, process design has developed rapidly across the globe to meet the demands of growing urban populations. Additionally, the role of wastewater treatment plants (WWTPs) has moved toward resource recovery as societies strive toward sustainability and a circular economy. However, the unsolved problems frequently experienced at the treatment plants are bulking and foaming resulting from overgrowth of filamentous bacteria, affecting stable operation, plant hydraulic capacity, and effluent quality.

Filamentous microorganisms are often linked to deteriorated sludge settleability and foam formation (Rossetti et al., 2005; Seviour and Nielsen, 2010; Wanner, 2017). A number of studies, mainly from Europe and northern China (Wang et al., 2016; Wanner, 2017; Fan et al., 2019), have identified species in the genus *Ca.* Microthrix as some of the most troublesome filamentous bacteria in municipal WWTPs in addition to *Gordonia*, *Ca.* Amarolinea, and other phylotypes belonging to the phylum Chloroflexi (Martins et al., 2004; Speirs et al., 2019; Nierychlo et al., 2020b). *Ca.* Microthrix has long been known by both researchers and practitioners in the field, and was identified almost 50 years ago (van Veen, 1973). It has been known as morphotype “Microthrix parvicella,” and can successfully be identified by light microscopy due to its distinct morphological features. These include unbranched, curled filaments with a hydrophobic surface staining Gram-positive, and often with clear polyphosphate (poly-P) granules visible after Neisser staining (van Veen, 1973; Eikelboom and van Buijsen, 1983; Nielsen et al., 2002). However, despite numerous studies on the physiology and ecology and potential control measures (Fan et al., 2020), a better understanding of its ecophysiology in activated sludge is needed to prevent *Ca.* Microthrix-related bulking and foaming events.

Two species of *Ca.* Microthrix are described in the literature: *Ca.* M. parvicella, abundant in municipal plants with nutrient removal (Erhart et al., 1997), and *Ca.* M. calida found in some industrial plants (Levantesi et al., 2006). Both species have been isolated and their physiology assessed in pure culture, although *Ca.* M. calida lacks a published reference genome. *Ca.* M. parvicella was shown to grow aerobically using volatile fatty acids (VFAs) or long chain fatty acids (LCFAs) (Slijkhuis, 1983; Tandoi et al., 1998), while *Ca.* M. calida is unable to grow on volatile fatty acids (VFAs) and requires LCFAs (Levantesi et al., 2006). However, the very slow growth of *Ca.* Microthrix isolates and difficulties with culture maintenance have hampered its extensive physiological characterization (Blackall et al., 1996; Fan et al., 2019).

Several *in situ* studies using fluorescence *in situ* hybridization (FISH) combined with microautoradiography or nano-scale secondary-ion mass spectrometry visualized important differences compared to the isolate studies. *Ca.* M. parvicella has never been observed *in situ* to consume VFAs but only LCFAs and glycerol (Andreasen and Nielsen, 1998; Kindaichi et al., 2013; Sheik et al., 2016). Furthermore, LCFA uptake followed by storage as lipids was observed under both aerobic and anaerobic conditions (Andreasen and Nielsen, 2000; Nielsen et al., 2002), which provides a competitive advantage in nutrient removal plants. *Ca.* M. parvicella were also shown to likely grow under anoxic conditions *in situ* by reducing nitrate to nitrite (Hesselsoe et al., 2005). The *in situ* activity of extracellular lipases (Schade and Lemmer, 2005) and their hydrophobic cell surface (Nielsen et al., 2002) further support the specialization of *Ca.* Microthrix spp. toward lipidic substrates. Presence of polyphosphate (poly-P) granules inside *Ca.* M. parvicella filaments has been shown in isolates and *in situ* (Erhart et al., 1997; Tandoi et al., 1998; Wang et al., 2014b; Fei et al., 2021; Petriglieri et al., 2021), placing it on the list of putative polyphosphate accumulating organisms (PAO). However, P cycling during alternating aerobic and anaerobic conditions has not been confirmed so far (Andreasen and Nielsen, 2000; Petriglieri et al., 2021). While the genome of *Ca.* M. calida has not been sequenced, two genomes representing *Ca.* M. parvicella are published: Dutch isolate Bio-17 (Muller et al., 2012), and Italian isolate RN1 (McIlroy et al., 2013). These genomes confirmed the physiology observed by *in situ* studies, showing assimilation and storage of LCFAs, the ability to store poly-P, and the potential to reduce nitrate and nitrite.

A few recent studies have made it possible to investigate the global diversity of *Ca.* Microthrix (Karst et al., 2018; Dueholm et al., 2020; Nierychlo et al., 2020a). The comprehensive and WWTP ecosystem-specific full-length 16S rRNA gene reference database, MiDAS4 (Dueholm et al., 2021), has enabled studies of the global WWTP microbiota at species-level resolution and provided unique placeholder names for the large diversity of undescribed populations. The ecosystem-specific database can also be used for the design and re-evaluation of FISH probes for the microbes of interest. These can be used in combination with other techniques to validate genomic information and confirm microorganism function in the WWTP ecosystem. Recent studies have granted valuable access to a high number of metagenome-assembled genomes (MAGs) representing microorganisms present *in situ* in the WWTP environment (Parks et al., 2017; Singleton et al., 2021). These MAGs together with high-throughput amplicon sequencing techniques provide a valuable tool set to explore the diversity of microbes present in the WWTP ecosystem.

In this study we investigated the diversity of genus *Ca.* Microthrix in activated sludge plants worldwide and found one abundant undescribed species: *Ca.* Microthrix subdominans. Using 16S rRNA gene amplicon sequencing data we investigated factors correlating to their global presence, and their influence on sludge settling properties. Furthermore, we compared the metabolic potential of *Ca.* M. parvicella and the *Ca.* M. subdominans based on new high-quality (HQ) MAGs. Both species were visualized *in situ* using novel FISH probes, which were further applied to investigate poly-P content using FISH-Raman microspectroscopy. Our results provide important new information about this troublesome filamentous genus in the activated sludge ecosystem, which may help to develop more targeted strategies for its control.

## Materials and Methods

### Biomass Sampling and Fixation and Sample Processing

Sampling of the activated sludge biomass from Danish full-scale municipal WWTPs was carried out within the MiDAS project (McIlroy et al., 2015; Nierychlo et al., 2020a). Sampling of full-scale plants across the world was coordinated by the MiDAS Global Consortium and carried out within the Global MiDAS project (Dueholm et al., 2021). Activated sludge was sampled from an aeration tank and sent directly to Aalborg University (Danish samples) or preserved in RNAlater and shipped to Aalborg University with cooling elements (Global samples). All samples were frozen upon arrival and stored until processing. For details, see Nierychlo et al. (2020a) and Dueholm et al. (2021).

### Amplicon Sequencing and Bioinformatic Analysis

DNA extraction, sample preparation, including amplification and amplicon sequencing were conducted as described in Nierychlo et al. (2020a) and Dueholm et al. (2021). Briefly, V1-V3 16S rRNA gene regions were amplified using the 27F (AGAGTTTGATCCTGGCTCAG) (Lane, 1991) and 534R (ATTACCGCGGCTGCTGG) (Muyzer et al., 1993) primers, and the resulting amplicons were used in all the analyses. The V4 16S rRNA gene region was amplified using the 515F (5′-GTGYCAGCMGCCGCGGTAA-3′) (Parada et al., 2016) and 806R (5′-GGACTACNVGGGTWTCTAAT-3′) (Apprill et al., 2015) primers. The Danish samples (Nierychlo et al., 2020a) were only sequenced with the V1-V3 primers, while the global samples (Dueholm et al., 2021) were sequenced by both primer pairs (V1-V3 and V4) to compare *Ca.* Microthrix abundances between the two amplicon datasets. The handling of the Danish and the global dataset in regard to DNA extraction and library preparation protocol were similar, providing comparable datasets. Raw fastq files were filtered for phiX sequences using usearch -filter_phix, trimmed to 250 bp using usearch -fastx_truncate -trunclen 250, and quality filtered using usearch -fastq_filter with -fastq_maxee 1.0. Dereplication was performed using usearch -fastx_uniques with -sizeout. Amplicon sequence variants (ASVs) were generated using -unoise3 (Edgar, 2016) with standard settings, and taxonomy was assigned using the MiDAS3 (for Danish dataset) or MiDAS4 (for Global dataset) reference database^1^ and the SINTAX classifier with -strand both and -sintax_cutoff 0.8 options (Edgar, 2018).

### Data Analysis and Visualization

R v.3.5.1 (R Core Team, 2020) and RStudio (RStudio Team, 2020) were used for data processing. The dataset with Danish plants contained 712 samples from 20 nutrient removal WWTPs with minimum 13,500 reads, the dataset with Global samples contained 847 samples from 438 WWTPs with 4 different process designs [carbon removal plants (C), carbon removal and nitrification (C,N), carbon removal, nitrification and denitrification (C,N,DN), and EBPR plants (C,N,DN,P)] with minimum 10,000 reads. All data was visualized using R packages ggplot2 v.3.2.1 (Wickham, 2009) and ampvis2 v.2.4.9 (Andersen et al., 2018). The Kruskal-Wallis test was used to determine statistically significant differences in *Ca.* Microthrix abundances across different groupings. Sequencing data for Danish and Global is available at the Sequence Read Archive^2^ as stated in Nierychlo et al. (2020a) and Dueholm et al. (2021), respectively.

### Phylogenetic Analysis of 16S rRNA Gene Sequences and Fluorescence *in situ* Hybridization (FISH) Probes Design

Phylogenetic analysis of 16S rRNA gene sequences and design of FISH probes for the novel species were performed using the MiDAS4 database containing high-quality full-length 16S rRNA gene sequences from the activated sludge ecosystem (Dueholm et al., 2021) using the ARB software v.6.0.6 (Ludwig et al., 2004). Novel probes were assessed *in silico* with the mathFISH software for hybridization efficiencies with target sequences, and potentially weak, non-target matches (Yilmaz et al., 2011). Where needed, unlabeled helper probes were designed to facilitate probe access to the target side. All probes were purchased from Biomers (Ulm, Germany), labeled with cyanine-3 (Cy3), cyanine-5 (Cy5) or 6-FAM fluorochromes.

A phylogenetic 16S rRNA gene tree was calculated based on full-length gene sequences retrieved from MiDAS4 (Dueholm et al., 2021) and SILVA 138 SSURef Nr99 (Quast et al., 2013) databases as well as gene sequences from the recovered MAGs using maximum likelihood method using the GTR model and 1000- replicates bootstrap analysis. Two of the MAGs had identical 16S rRNA gene sequences (Bjer_MB2 and AalW_MB3), so one was chosen for incorporation into the Figure 4 tree, which is labeled to indicate this. Trees were viewed in ARB v6.0.3 (Ludwig et al., 2004), and displayed in iTOL v5.7 (Letunic and Bork, 2019) with final presentation in Inkscape v0.92.

### FISH, Probe Optimization, and Quantitative FISH (qFISH)

FISH was performed as previously described (Nielsen, 2009). Validation and optimization of novel probes were based on formamide (FA) dissociation curves by carrying out hybridizations over a range of 0–70% (v/v) FA concentrations with increments of 5% (data not shown). Activated sludge samples from Danish WWTPs with high abundance of the target organism, as predicted by amplicon sequencing, were used. Microscopic analysis was performed with Axioskop epifluorescence microscope (Carl Zeiss, Germany) equipped with LEICA DFC7000 T CCD camera or with a white light laser confocal microscope (Leica TCS SP8 X). The intensity of at least 50 cells at each FA concentration was measured with the ImageJ software (National Institutes of Health, Bethesda, MD, United States). Optimal hybridization conditions and details about the coverage and specificity of all the probes used in the study are presented in Table 1. EUBmix (Amann et al., 1990; Daims et al., 1999) was used to target all bacteria. The NON-EUB probe (Wallner et al., 1993) was used as a negative control for sequence independent probe binding. Quantitative FISH (qFISH) biovolume fractions of individual species and genera were calculated as a percentage area of the total biovolume, hybridizing with both EUBmix and specific probes. qFISH analysis was performed using the Daime image analysis software (Daims et al., 2006), based on 30 fields of view taken at 630× magnification.

**TABLE 1:** Overview of the existing and new FISH probes targeting *Ca.* Microthrix.

Probe	*E. coli* pos.	Target group	Coverage*	Non-target hits	Sequence (5′–3′)	Pre-treatment**	%FA***	References
MCX840	840–859	Genus *Ca.* Microthrix	MiDAS3: 8/14 MiDAS4: 21/28 Silva138: 24/28	0 1 0	CGG CGC GGA GAG AGT TGA GT	A	20	This study
MCX840_H1	–	Helper for MCX840			TCT CCC CAC ACC TAG TGC CCA ACG		–	
MCX840_H2	–	Helper for MCX840			GCG GGG CAC TTA ATG CGT TAG CTA		–	
Mpa177	177–199	*Ca.* Microthrix parvicella	MiDAS3: 3/3 MiDAS4: 4/6 Silva138: 11/28	0 0 1	GCG GTG AAG AGA AGG TAT GCG GT	A	45	This study
Mpa177_H1	–	Helper for Mpa177			AGA GCG ATA AAT CTT TCT TCA ACT CAC CAT		–	
Mpa177_H2	–	Helper for Mpa177			TCG AAT TTC TTC GAG TTA TTC CCC ACT CC		–	
Msu181	181–203	*Ca.* Microthrix subdominans	MiDAS3: 2/4 MiDAS4: 4/10 Silva138: 8/28	0 0 0	CCA CCA TTC GAC GGT AAG AAG GT	A	40	This study
Msu181_H1	–	Helper for Msu181			CTC CCA GAG CGA TAA ATC TTT CTT CAA A		–	
Msu181_H2	–	Helper for Msu181			ATG CGG TAT TAG CTC GAA TTT CTT CGA		–	
MPA60	60–77	*Ca.* Microthrix parvicella	MiDAS3: 3/3 MiDAS4: 6/6 Silva138: 8/28	0 0 1	GGA TGG CCG CGT TCG ACT	B/C/D	20	Erhart et al., 1997
MPA223	223–240	Genus *Ca.* Microthrix	MiDAS3: 4/14 MiDAS4: 10/28 Silva138: 9/28	8 20 28	GCC GCG AGA CCC TCC TAG	B/C/D	20	Erhart et al., 1997
MPA645	546–661	Genus *Ca.* Microthrix	MiDAS3: 14/14 MiDAS4: 27/28 Silva138: 27/28	0 1 1	CCG GAC TCT AGT CAG AGC	B/C/D	20	Erhart et al., 1997
MPA650	650–666	Genus *Ca.* Microthrix	MiDAS3: 14/14 MiDAS4: 28/28 Silva138: 27/28	41 103 151	CCC TAC CGG ACT CTA GTC	B/C/D	20	Erhart et al., 1997
Mpa_all_ 1410	1411–1430	*Ca.* Microthrix calida	MiDAS3: 5/6 MiDAS4: 6/7 Silva138: 1/1	1 1 0	GGT GTT GTC GAC TTT CGG CG	B/C/D	35	Levantesi et al., 2006
Mpa-T1- 1260	1261–1280	*Ca.* Microthrix calida	MiDAS3: 6/6 MiDAS4: 7/7 Silva138: 1/1	0 1 0	TTC GCA TGA CCT CAC GGT TT	B/C/D	25	Levantesi et al., 2006

*^∗^Probe coverage is given for MiDAS 3.7 (Nierychlo et al., 2020a), 4.8 (Dueholm et al., 2021), and Silva 138 (Quast et al., 2013) database. Values given as group hits/group totals; ^∗∗^A: 30 min lysozyme followed by 30 min achromopeptidase and overnight hybridization; B: 15–25 min mutanolysin (Erhart et al., 1997); C: achromopeptidase and lysozyme (Kragelund et al., 2007); D: lysozyme and extended hybridization time 15–18 h; ^∗∗∗^Recommended optimal FA concentration for use in FISH hybridizations.*

### Morphological Characterization

A minimum of 60 probe-defined filaments were randomly chosen to measure cell length and width using the segmented line tool in ImageJ (National Institutes of Health, Maryland United States).

### Raman Microspectroscopy

Fresh activated sludge samples were collected from full-scale Danish WWTPs and aerated for 30 min to exhaust most intracellular carbon and refill poly-P reserves. Samples were fixed in 50% ethanol (final concentration), as previously described (Nielsen, 2009), and stored at –20°C until analysis. Raman microspectroscopy was applied in combination with FISH as previously described (Fernando et al., 2019). Briefly, FISH was conducted on optically polished CaF_2_ Raman windows (Crystran, United Kingdom). The specific FISH probes were used to locate the target cells with a 50X dry objective (Olympus M Plan Achromat- Japan) of the in-built Olympus (model BX-41) fluorescence microscope. After bleaching, spectra from different filaments were obtained using a Horiba LabRam HR 800 Evolution (Jobin Yvon—France) equipped with a Torus MPC 3000 (United Kingdom) 532 nm 341 mW solid-state semiconductor laser. The specific settings for the spectrophotometer were: 5% neutral density (ND) filters, 600 mm/groove diffraction grating, 100 μm and 72 μm slidth width and confocal pinhole, respectively. The Raman spectra collected spanned the wavenumber region of 200 cm^–1^ to 3,000 cm^–1^. The Raman spectrometer was calibrated prior to obtaining all measurements to the first-order Raman signal of Silicon, occurring at 520.7 cm^–1^. Raman spectrometer operation and subsequent processing of spectra were conducted using LabSpec version 6.4 software (Horiba Scientific, France). Absolute quantification of intracellular poly-P was carried out as described by Fernando et al. (2019). The method assumes that the intensity of the Raman signal is directly dependent on the amount of the analyte in a determined area. An average amount of poly-P per cell was calculated as a factor of a constant determined during calibration for poly-P (Fernando et al., 2019), the average charge-coupled device (CCD) counts determined during the experiment, and the average area of cells measured by image analysis. For filaments, it is very difficult to determine the internal area of single cells with common microscopic techniques. An arbitrary area was determined by multiplying the filament width by 1 μm, thus obtaining the amount of poly-P in an average filament segment of 1 μm length.

### Identification, Annotation, and Metabolic Reconstruction of *Ca*. Microthrix

*Ca.* Microthrix MAGs were identified from within a set of 1083 high-quality MAGs from Danish activated sludge WWTPs (Singleton et al., 2021, NCBI BioProject PRJNA629478). Ten MAGs were identified, and followed the MIMAG standard for high-quality draft genomes, including full-length rRNA genes, and a completeness > 90%, and contamination < 5%. GTDB-Tk v1.4.1 (Chaumeil et al., 2020) with RefSeq release 95 was used for taxonomic classification, and the species representatives were selected using dRep v2.3.2 (Olm et al., 2017) at 95% average nucleotide identity and the CheckM v1.1.2 (Parks et al., 2015) completeness and contamination quality statistics. 16S rRNA genes in the MAGs were identified within the genomes using Infernal v1.1.2 (Nawrocki and Eddy, 2013), and BEDTools v2.27 (Quinlan and Hall, 2010) was used to extract the sequences for inclusion in the 16S rRNA gene phylogenetic tree. *Ca.* M. parvicella genomes and MAGs present in NCBI but not the dereplicated GTDB-Tk Refseq release 95 database were downloaded (on 26/02/2021) and incorporated with the Danish MAGs and the GTDB-Tk set for phylogenomic analysis. The genome tree was created from the concatenated, trimmed alignment of 120 single copy proteins produced by GTDB-Tk. IQ-TREE v2.0 (Nguyen et al., 2015) was used to create a maximum likelihood tree from this alignment following the WAG+G model with 100 bootstrap iterations. Trees were viewed in ARB v6.0.3 (Ludwig et al., 2004), and displayed in iTOL v5.7 (Letunic and Bork, 2019) with final presentation in Inkscape v0.92.

EnrichM v0.5.0^3^ “annotate” was used to annotate the MAGs against the KEGG Orthology (KO) number (Kanehisa and Goto, 2000) annotated uniref100 database (EnrichM database v10) using the default settings. EnrichM “enrichment” was used to determine the enriched presence of KOs in the *Ca.* M. parvicella or *Ca.* M. subdominans MAGs, to determine species differences. Additionally, the MAGs were uploaded to the “MicroScope Microbial Genome Annotation and Analysis Platform” (Vallenet et al., 2020) in order to examine gene synteny and cross-validate KO annotations found using EnrichM. Core and species specific genes were also examined using the MicroScope platform (Vallenet et al., 2020). Specifically, the *Ca.* M. subdominans MAGs were compared to the *Ca.* M. parvicella MAGs and the *Ca.* M. parvicella RN1 isolate genome using the pan-genome analysis function using 50% amino acid identity and 80% alignment coverage. Core genes specific to each species were identified and examined within the surrounding genomic context (i.e., syntenic genes), and additional checks were conducted using protein BLAST against the NCBI nr database (Camacho et al., 2009).

Average nucleotide identity based on blast (ANIb) was conducted using pyani v0.2.10 (Pritchard et al., 2016) and the “average_nucleotide_identity.py -m ANIb” arguments. R v4.0.3 (R Core Team, 2020), and the libraries gplots, RColorBrewer and reshape2, were used to process the produced files, ANIb_percentage_identity.tab and ANIb_alignment_coverage.tab, into ANI and alignment heatmaps with the heatmap.2 function. Inkscape v0.92 was used for the final presentation.

## Results and Discussion

### Diversity and Distribution of *Ca*. Microthrix Species in Full-Scale WWTPs by Amplicon Sequencing

**FIGURE 1:**
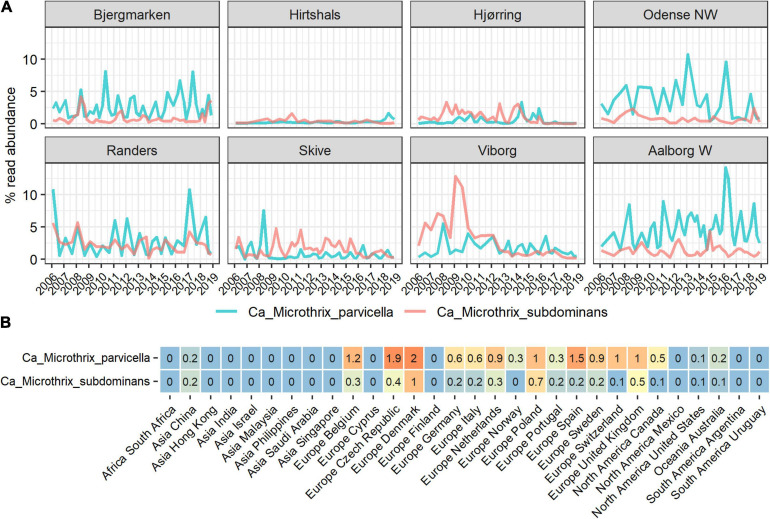
Abundance of *Ca.* Microthrix species in full-scale activated sludge plants in **(A)** Denmark and **(B)** globally, shown by country. The Danish data **(A)** represents a subset of the long-term survey of microbial communities in years 2006–2018 (Nierychlo et al., 2020a) containing 311 samples from 8 nutrient removal WWTPs. The global data **(B)** represents average abundances per country of a total of 847 activated sludge samples from 438 plants with four different process designs (carbon removal, carbon removal and nitrification, carbon removal, nitrification and denitrification, and EBPR plants).

The diversity and occurrence of *Ca.* Microthrix species in the activated sludge ecosystem were analyzed using data from long-term surveys of Danish WWTPs (Nierychlo et al., 2020a) as well as the recent Global WWTP survey of 438 samples (Dueholm et al., 2021). We observed a widespread presence of two *Ca.* Microthrix species (Figure 1): the well-known and characterized *Ca.* M. parvicella, and a second, novel species with the provisional name in the MiDAS taxonomy: midas_s_2, but here given the name *Candidatus* Microthrix subdominans (see section Etymology). The two other low-abundant species were not observed as abundant in any WWTP. *Ca.* M. parvicella was dominant in the majority of the Danish and global plants, with the average read abundance of 1.7 ± 2.0% and 0.6 ± 1.2%, respectively. In some plants their abundance exceeded 14% likely causing severe sludge separation problems and deteriorated plant performance. *Ca.* M. subdominans was generally observed at lower abundances, with average and maximum read abundances in Denmark of 1.1 ± 1.3 and 13%, respectively, and average and maximum abundances globally of 0.2 ± 0.5 and 4%, respectively. Both species co-existed in the majority of the plants, indicating a similar ecological niche, with the highest global abundances recorded in a number of European countries (Figure 1B). The only other known *Ca.* Microthrix species, *Ca.* M. calida, previously isolated from industrial WWTP (Levantesi et al., 2006), was not observed in any of the plants investigated.

**FIGURE 2:**
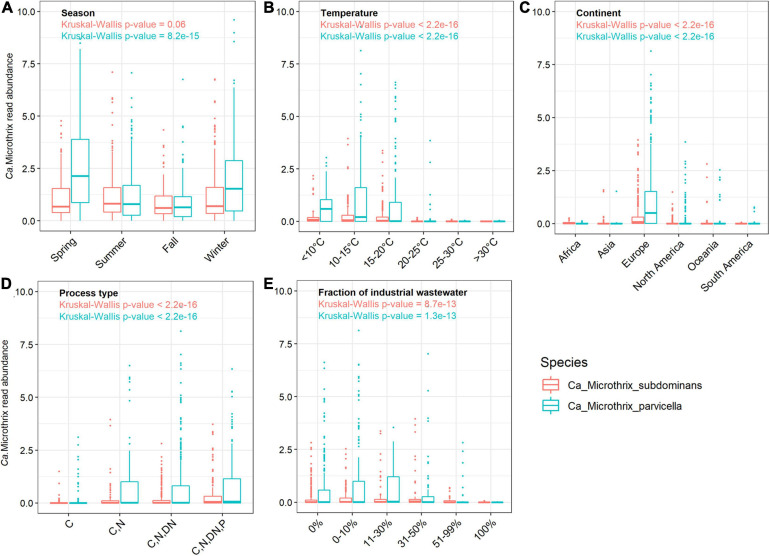
Occurrence of *Ca.* Microthrix species across **(A)** different seasons in Denmark, and across the world in relation to **(B)** temperature **(C)** continents, **(D)** process types, and **(E)** in WWTPs with different fractions of industrial wastewater. The non-parametric Kruskal-Wallis test was used to determine the statistical support for differences between the means of the groupings. The Danish dataset containing 712 samples from 20 WWTPs sampled in the period 2006–2018 (Nierychlo et al., 2020a) was used to visualize seasonal differences in *Ca.* Microthrix species occurrence.

In the Danish nutrient removal plants *Ca.* M. parvicella was the second most abundant genus (Supplementary Figure 1), underlining its importance for wastewater treatment processes and floc properties. Long-term survey of Danish plants provided insight into the abundance levels and seasonal dynamics of the two species (Figure 1A). In some plants *Ca.* Microthrix was hardly present, in others consistently found over many years. *Ca.* M. parvicella was dominant in most plants. The plants have only minor differences in design and operation, so the mechanisms behind the observed differences are not known. The survey revealed clear seasonal pattern across the plants, visible in particular for *Ca.* M. parvicella (Figures 1A, 2A), with the highest abundance observed during winter (sampled in February) and spring (sampled in May). This is in accordance with earlier observations of its excessive proliferation and bulking in colder seasons (e.g., Kruit et al., 2002; Hug et al., 2006; Mielczarek et al., 2012). Interestingly, *Ca.* M. subdominans did not show a strong seasonal pattern, and had no statistical support in the Danish dataset for differential abundance across the seasons (*p* = 0.06).

The comprehensive global dataset of WWTP community composition enabled a deeper insight into factors affecting the occurrence of the two *Ca.* Microthrix species (Figures 2B–E). Temperature showed a strong effect on the occurrence of both species, with highest abundance observed in WWTPs below 15°C and hardly any *Ca.* Microthrix found in plants with temperature exceeding 20°C (Figure 2B). This observation confirms the general notion that *Ca.* Microthrix thrives at lower temperatures (Knoop and Kunst, 1998; Sheik et al., 2016), which is also reflected by its geographical distribution with the highest abundances of both species observed in Europe (Figure 2C). The process design was important for the prevalence of *Ca.* Microthrix, with highest abundances of both species in more advanced plants with biological N and P removal (Figure 2D). This supports previous observations of *Ca.* Microthrix preferentially occurring in plants with long sludge retention time (SRT) and aerobic and anoxic stages (Noutsopoulos et al., 2007; Fan et al., 2020) reflecting its low growth rate, the ability to store lipid under anaerobic conditions, and use of nitrate as electron acceptor (Rossetti et al., 2005). The fraction of industrial wastewater, here given as the COD fraction in the influent, was also shown to be important for the occurrence of *Ca.* Microthrix (Figure 2E). Both species were primarily found in municipal plants with low to medium content of industrial wastewater. The reason may be that industrial WWTPs rarely have a high content of lipidic substrates in the influent, which is an important factor promoting growth of *Ca.* Microthrix.

**FIGURE 3:**
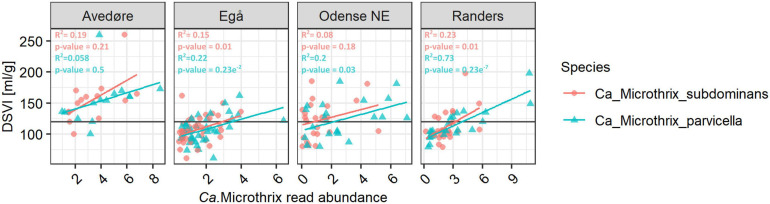
Relationship between *Ca.* Microthrix species abundance and DSVI in chosen full-scale Danish nutrient removal plants. The Danish data subset containing 128 samples from 4 WWTPs sampled in the period 2006–2018 (Nierychlo et al., 2020a) was used. Values above the line of 120 ml/gSS for DSVI represent sludge with poor settling properties. To determine the statistical support of *Ca.* Microthrix species abundance relationship with DSVI, ANOVA test was used.

Both species had substantial effects on the settling properties in Danish WWTPs. The diluted sludge volume index (DSVI), which is widely used as a measure of sludge settling properties, showed a positive relationship with the relative abundance of both *Ca.* Microthrix species (Figure 3). Such relationships have been demonstrated before (e.g., Wang et al., 2014a,b; Fan et al., 2018; Nierychlo et al., 2020b) either for the whole genus or for *Ca.* M. parvicella. With the new species-specific FISH probes (see the next section), we could observe that in plants where both species were abundant, they had a similar effect on sludge settling. This was visualized by an almost identical slope representing the relationship between species read abundance and the corresponding DSVI values (Figure 3).

### Phylogenetic Analysis and Visualization of *Ca*. Microthrix Species by FISH

**FIGURE 4:**
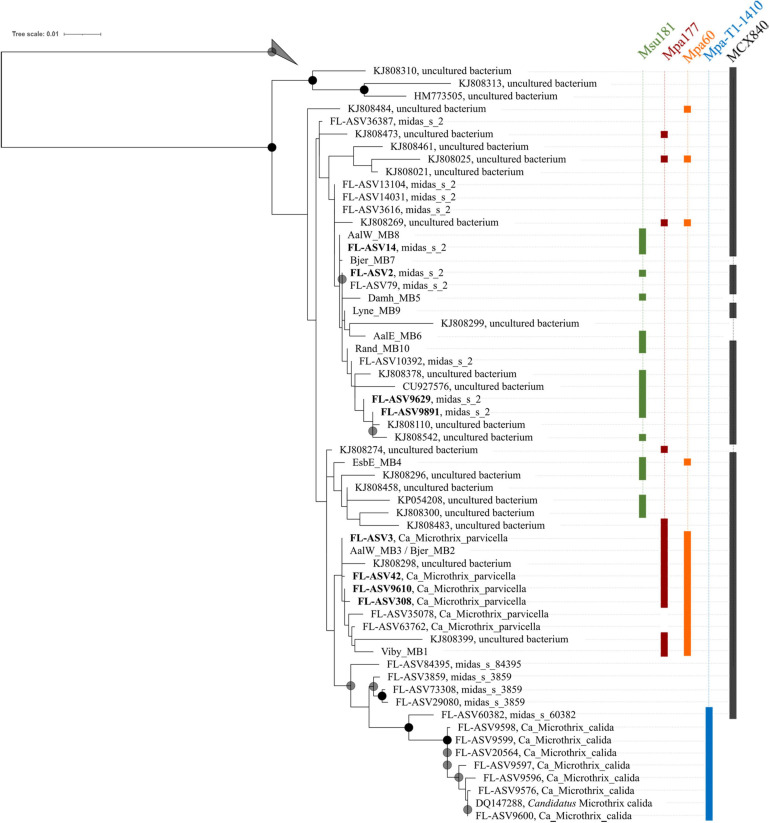
Maximum-likelihood (PhyML) 16S rRNA gene phylogenetic tree of the genus *Ca.* Microthrix and coverage of new and existing FISH probes recommended for use. The tree includes full-length sequences from MiDAS4 database (FL-ASVxxx), MAG (xxxx_MBx_x) and chosen SILVA138 SSURef Nr99 (indicated by sequence accession numbers). FL-ASVs corresponding to the globally abundant ASVs are shown in bold. FL-ASVs representing *Ca.* M. subdominans are shown with its provisional name midas_s_2 assigned by MiDAS4.8 taxonomy. Coverage of individual FISH probes is shown in panels to the right. A 20% conservational filter was applied to the alignment used for the tree to remove hypervariable positions, resulting in 1204 aligned positions. Bootstrap values from 1000 re-samplings are indicated for branches with > 70% (gray circle), and > 90% (black circle) support. 4 FL-ASVs representing a novel genus from the order IMCC26256 were used as the outgroup. The scale bar represents substitutions per position.

Phylogenetic analysis of the genus *Ca.* Microthrix was conducted using full-length 16S rRNA gene sequences obtained from the MiDAS4 database, SILVA 138 SSURef Nr99, and from MAGs recovered from Danish WWTPs (Figure 4). *Ca.* Microthrix is represented by 6 distinct species according to the MiDAS4 taxonomy: parvicella, calida, and several species with provisional midas_s_x names, including midas_s_2 (named here *Ca.* Microthrix subdominans). However, only two of the species were abundant globally in WWTPs (Figure 1): *Ca.* M. parvicella and *Ca.* M. subdominans, the latter without a cultured representative or genome.

The first FISH probes targeting morphotype *Microthrix parvicella* were designed more than 20 years ago (Erhart et al., 1997), and additional probes were designed by Levantesi et al. (2006) after isolation of *Ca.* M. calida. Their coverage and specificity for targeting individual *Ca.* Microthrix species were tested *in silico* using the MiDAS4 reference database (Table 1) in addition to new probes designed in this study. Probe MPA60 was originally designed to visualize the *M. parvicella* morphotype, and it targets species *Ca.* M. parvicella with perfect coverage and specificity. Probes MPA223 and MPA650 have low specificity, so their use in activated sludge is not recommended. The probe MPA645 served as a good candidate for a genus-level probe, but *in silico* testing and *in situ* hybridization directly in activated sludge revealed sub-optimal binding resulting in weak FISH signal (Supplementary Figure 2A). The *in silico* test of probe Mpa-T1-1260 to target *Ca.* M. calida (Levantesi et al., 2006) indicated good coverage and specificity, but the use of probe Mpa_all_1410 as a general genus-level probe cannot be recommended due to insufficient coverage.

**FIGURE 5:**
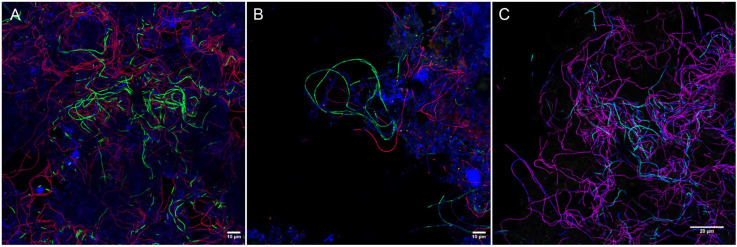
Composite FISH micrographs of *Ca.* Microthrix species in full-scale activated sludge. **(A)** *Ca.* M. parvicella visualized with species-specific probes Mpa177 (Cy3, red), *Ca.* M. subdominans visualized with probe Msu181 (6-FAM, green), other bacteria shown with EUBmix (Cy5, blue); **(B)** *Ca.* M. parvicella visualized with species-specific probe MPA60 (Cy3, red), *Ca.* M. subdominans visualized with probe Msu181 (6-FAM, green), other bacteria shown with EUBmix (Cy5, blue); **(C)** *Ca.* M. parvicella visualized with species-specific probes Mpa177 (Cy3, red) appear magenta, *Ca.* M. subdominans visualized with probe Msu181 (6-FAM, cyan) appear white, *Ca.* Microthrix genus visualized with probe MCX840 (Cy5, blue). Activated sludge was sampled from Randers WWTP.

None of the existing probes targeted the novel *Ca.* M. subdominans, and the general genus-level probes were of insufficient quality, thus three new probes: two species-specific probes and one genus-level probe, were designed and optimized (Table 1). Since the existing probe MPA60 with 1 mm targets the majority of midas_s_2 sequences, an alternative species-specific probe for *Ca.* M. parvicella was designed. When applied to full-scale activated sludge biomass, all three new probes exclusively hybridized with curly filaments entangled inside, as well as protruding from, the flocs (Figure 5). *Ca.* M. parvicella filaments visualized with probe Mpa177 (Figure 5A) were 0.7 ± 0.1 μm wide and 122 ± 11 μm long, and *Ca.* M. subdominans visualized with probe Msu181 (Figures 5A,B) were 1.0 ± 0.2 μm wide and 92 ± 19 μm long. No overlap between the two species-specific probes was observed, confirming their specificity *in situ*, and both showed a good overlap with the genus-level probe MCX840 (Figure 5C). Probe Mpa177 targeting *Ca.* M. parvicella ensures high specificity as it does not target other *Ca.* Microthrix species even with one mismatch. However, *in situ* tests performed using the existing *Ca.* M. parvicella probe MPA60 also proved its high specificity, without overlap with *Ca.* M. subdominans (Figure 5B). Mpa177 and MPA60 showed very good overlap (Supplementary Figure 2B), thus both probes can be applied interchangeably, or with different fluorochromes to confirm specific coverage of *Ca.* M. parvicella, or together as a mix to give higher fluorescence signal. Helper probes are recommended with all the new probes to ensure strong and even fluorescence signal along the filaments.

Quantitative FISH analyses with all new probes showed that the biovolume fractions of the biomass were similar or slightly higher than the read abundance by amplicon sequencing using the V1-V3 primers (Table 2). The amplicon abundance estimation for *Ca.* Microthrix depended strongly on primers used, with notable divergence between the two most commonly used primer sets in microbial community analyses. We observed that *Ca.* Microthrix abundance is underestimated by the factor of 3 by the V4 primer set, compared to V1-V3 primer set (Supplementary Figure 3). This is of importance when comparing abundance estimates by the two methods and when the potential effect on settling properties is evaluated using amplicon sequencing data.

**TABLE 2:** Ca. Microthrix abundance estimation using amplicon sequencing and qFISH.

FISH Probe (target) WWTP		Abundance [%]	
	Date	Amplicon reads*	qFISH
**Mpa177 (*Ca*. M. parvicella)**			
*Randers*	February 2017	10.9	19.3 (±5.9)
*Viborg*	February 2010	3.6	5.0 (±2.0)
*Kolding*	February 2008	7	8.9 (±4.8)
**Msu181 (*Ca.* M. subdominans)**			
Randers	February 2017	4.2	5.8 (±2.2)
Viborg	February 2010	4.3	4.9 (±1.3)
Kolding	February 2008	4.2	4.2 (±2.5)
**MCX840 (genus *Ca.* Microthrix)**			
Randers	February 2017	15.1	22.9 (±7.0)
Viborg	February 2010	7.9	15.5 (±3.5)
Kolding	February 2008	11.2	16.2 (±6.1)

*Standard deviation of amplicon sequencing is approx. 10%.

High sequence similarity among the species in genus *Ca.* Microthrix makes it difficult to construct robust phylogenetic trees, resulting in low bootstrap values (Figure 4). Consequently, the exact placement of the individual sequences, especially those clustering with the two most abundant species, may not be definitive. Not all full-length 16S rRNA gene sequences coming from the MAGs or retrieved from the SILVA database are targeted by the new or existing FISH probes (e.g., the MAG Bjer_MB7 representing *Ca.* M. subdominans or KJ808299). However, it should be kept in mind that the MAGs recovered may not always represent the abundant members of the microbial community, and sequences retrieved from SILVA are clustered at 97% similarity, both of which may result in the *in silico* observed lack of probe coverage. All the MAG-derived sequences clustered either with *Ca.* M. parvicella or *Ca.* M. subdominans, except EsbE_MB4, which formed a separate cluster with several SILVA sequences. This particular sequence was targeted by *Ca.* M. parvicella-specific probe (MPA60) and the *Ca.* M. subdominans-specific probe (Msu181).

### Detection and Quantification of Storage Polymers in *Ca*. Microthrix

**FIGURE 6:**
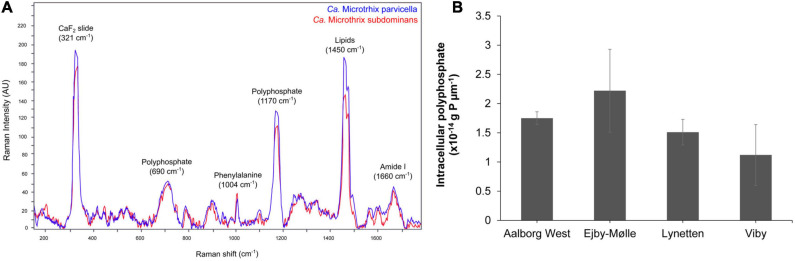
FISH-Raman analysis of *Ca.* Microthrix filaments. **(A)** Raman spectra showing the presence of poly-P as well as lipidic compound in both *Ca.* Microthrix species, **(B)** quantification of poly-P content in *Ca.* Microthrix filaments (at the genus-level) in activated sludge sampled from a full-scale plant aeration tank (adapted from Petriglieri et al., 2021).

Application of the species-specific FISH probes with Raman microspectroscopy revealed the presence of biological peaks for nucleic acids (784 cm^–1^), phenylalanine (1,004 cm^–1^), lipids (1,450 cm^–1^), amide I linkages of proteins (1,660 cm^–1^), as well as poly-P storage (1,170 cm^–1^) (Schuster et al., 2000; Huang et al., 2004) in both *Ca.* M. parvicella and *Ca.* M. subdominans (Figure 6A). No other storage polymers pertinent to the PAO metabolism (glycogen or PHA) were detected *in situ* under aerobic or anaerobic conditions. Interestingly, the peak assigned to lipids (1,450 cm^–1^) was significantly higher than similar peaks observed in other PAOs (Fernando et al., 2019), supporting the prevalence of lipid metabolism suggested by the metabolic potential.

The amount of stored poly-P in *Ca.* Microthrix filaments was quantified using FISH-Raman microspectroscopy in fresh activated sludge from four full-scale EBPR plants (Figure 6B). The content of poly-P varied slightly among the different plants, with the highest value measured in Ejby-Mølle (2.22^∗^10^–14^ g P μm^–1^) and the lowest in Viby (1.12^∗^10^–14^ g P μm^–1^). Even though the *Ca.* Microthrix trichome width was relatively small, and thus also the P-content per μm of the filament, it can substantially contribute to P removal in the plants as the filament length often exceeds 100 μm, and the genus often present at high abundances (Petriglieri et al., 2021).

In order to investigate whether *Ca.* Microthrix had a dynamic poly-P uptake and release in anaerobic- aerobic cycles as are typical in EBPR plants, we carried out a number of experiments with fresh activated sludge with a high content of *Ca.* M. parvicella and *Ca.* M. subdominans (Supplementary Note 2 and Supplementary Table 3). None of the substrates tested (mixture acetate-glucose-casamino acids or oleic acid) induced anaerobic depletion of intracellular poly-P after 3 h without oxygen or nitrate present. This supports previous observations (Andreasen and Nielsen, 2000) that *Ca.* Microthrix does not have a dynamic P-cycling similar to PAOs.

### *Ca*. Microthrix Genome Recovery and Metabolic Reconstruction

**FIGURE 7:**
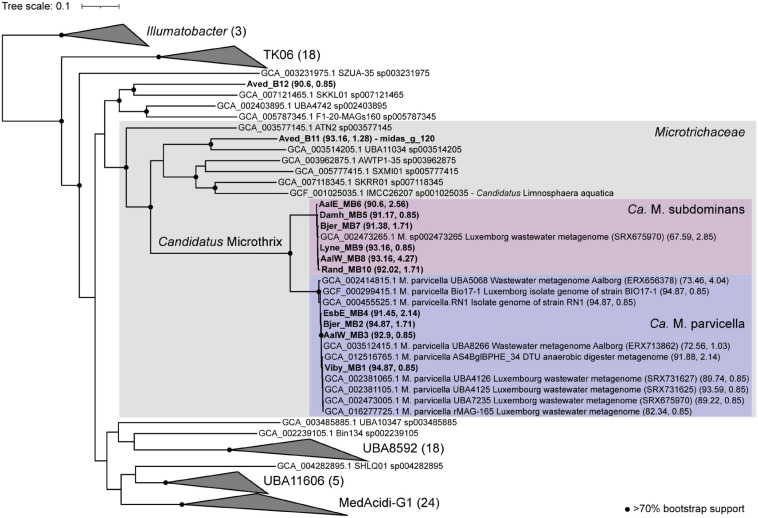
Phylogenetic maximum likelihood genome tree of the *Ca.* Microthrix MAGs and related genomes using the concatenated alignment of 120 single copy proteins created by GTDB-Tk and the GTDB RefSeq release 95. Three *Illumatobacter* isolate genomes were used as the outgroup. Bootstrap support > 70% is shown by the solid black circles.

Eleven high-quality MAGs from the family Microtrichaceae were recovered from across 23 Danish WWTPs (Singleton et al., 2021). Ten of these MAGs were from the genus *Ca.* Microthrix, forming two different species (95% ANI clustering), *Ca.* M. parvicella (4 MAGs) and *Ca.* M. subdominans (6 MAGs) (Figure 7). One MAG (Aved_B11) belonged to a novel and undescribed genus with the provisional name midas_g_120. These two genera, as well as genus IMCC26207 represented by the genome of *Candidatus* Limnosphaera aquatica strain IMCC26207 (Kim et al., 2017), were found to be abundant across global WWTPs (Supplementary Figure 4).

The number of coding sequence (CDS) regions for *Ca.* Microthrix MAGs ranged between 3915 and 4743, and completeness and contamination ranged between 90.6–94.87 and 0.85–4.27%, respectively (Supplementary Data 1). To date, only one medium quality MAG (GCA_002473265, 62% completeness), recovered from a WWTP metagenome in Luxembourg (SAMN02862044), exists for *Ca.* M. subdominans in the GTDB database (Parks et al., 2017). The four *Ca.* M. parvicella MAGs are > 99% ANI to each other (alignment coverage > 79%; Supplementary Figure 5), representing near identical lineages despite being recovered from separate WWTPs. The six *Ca.* M. subdominans MAGs had ANIs of > 98% (alignment coverage > 74%) to each other, suggesting a similar lack of diversity (Supplementary Figure 5). This supports previous findings suggesting lower genomic diversity between *Ca.* Microthrix lineages when compared to other common genera in WWTPs, such as *Ca.* Accumulibacter (McIlroy et al., 2013). Additionally, the analyses of single-nucleotide polymorphisms in *Ca.* Microthrix spp. has indicated low population-level diversity (Muller et al., 2014a).

**FIGURE 8:**
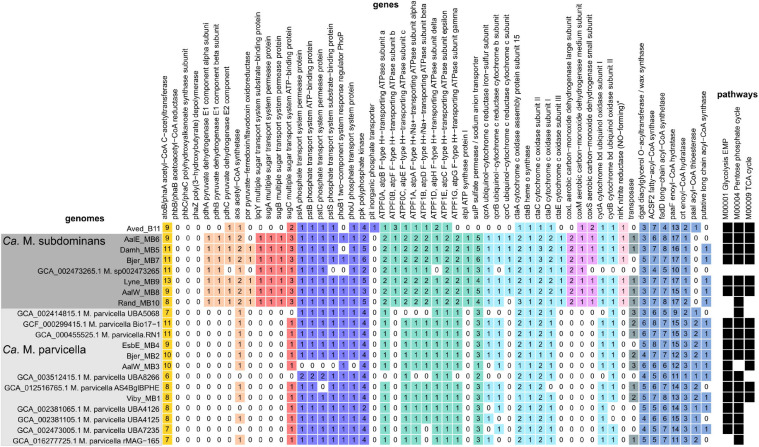
Presence, absence and copy number of genes in the *Ca.* M. parvicella and *Ca.* M. subdominans MAGs and genomes discussed in this study. Genes were detected using the KO analysis, with the specific KOs shown in Supplementary Data 2. The asterisk for *nirK* indicates that this gene was only detected in *Ca.* M. subdominans using this approach, but manual inspection of the *Ca.* M. parvicella RN1 genome in MAGE revealed a putative *nirK*, as found previously (McIlroy et al., 2013). The pathways indicate the KEGG modules with at least 80% completeness (black squares) for metabolisms described in the text (Supplementary Data 3).

As expected based on the high similarity of the *Ca.* M. parvicella MAGs to the previously recovered genomes, metabolic reconstruction revealed similar metabolisms to the published models. The *Ca.* M. parvicella MAGs encoded the EMP pathway, TCA cycle, pentose phosphate pathway, as well as lipid activation and oxidation capabilities, high-affinity phosphate transporters, potential triacylglycerol (TAG) storage, and the potential to reduce nitrite and nitrate (McIlroy et al., 2013). Unlike *Ca.* M. parvicella, the metabolic potential of *Ca.* M. subdominans is currently unknown. The pangenome of *Ca.* Microthrix based on the orthologous proteins shared between the two species was large (11,765 gene families, Supplementary Table 1). However, the core genome of 1,513 gene families showed similar metabolic capabilities to *Ca.* M. parvicella. Numerous acyl-CoA ligases (K00666, K01897, K03822, up to 8 copies per genome) and enoyl-CoA hydratases (K01692, K01715, up to 17 copies per genome) were detected in every MAG, supporting lipid use as a key metabolic strategy (Figure 8). Diacylglycerol O-acyltransferase / wax synthase (K00635, Figure 8) genes (3–5 copies per MAG) were identified in the *Ca.* M. subdominans MAGs, supporting the hypothesized use of TAG as an energy storage compound (McIlroy et al., 2013). While PHB storage and use has been suggested in previous work (Muller et al., 2012), we could only identify copies of the *phaA* gene. No copies of *phaB* or the key gene, PHA synthase (*phaC*), were detected in the KO analysis (Figure 8). Absence of these genes supports the Raman microspectroscopy analyses above showing no PHA storage. The potential for nitrate (putative genes identified in McIlroy et al., 2013) and nitrite reduction (*nirK*, K00368) in anaerobic respiration was also identified in *Ca.* M. subdominans (Figure 8). Genes for further reduction to N_2_O or N_2_ were not detected. The phosphate metabolism genes were highly similar between the two species. Genes for the high affinity phosphate transporter (*pstSCAB*) and transport regulator (*phoU*) were detected in *Ca.* M. subdominans (Figure 8). Similar to *Ca.* M. parvicella, the low affinity Pit transport system, often used as a likely indicator gene for polyphosphate accumulating organisms such as *Tetrasphaera* and *Ca.* Accumulibacter (McIlroy et al., 2014; Oyserman et al., 2016), was not detected. No acetate transporter genes were identified in the genomes, though genes for the transport of branched chain amino acids were detected (*livGFHMK*, K01995-K01999). While the main metabolisms were very similar between the two species, a subset of genes comprised the species-specific core metabolisms of *Ca.* M. parvicella (380) and *Ca.* M. subdominans (364) (Supplementary Table 1).

Genes unique to *Ca.* M. subdominans indicated a slightly different niche to *Ca.* M. parvicella. Firstly, this lineage may be able to use atmospheric levels of carbon monoxide as a supplementary energy source, as it appears to encode several genes for a putative form II aerobic carbon monoxide dehydrogenase (CODH, K03518. K03519, K03520, *coxLMS*) (Figure 8; Supplementary Note 1). This could be an adaptation of *Ca.* M. subdominans to its upstream habitats, most likely soil and water systems (King, 2003; King and Weber, 2007). An ABC sugar transporter unique to *Ca.* M. subdominans was also identified (Figure 8). The four genes comprising the ABC transporter operon (*lpqY*, *sugABC*, K02027, K02025, K02026, K10112) are most closely related to those found in the ammonia oxidizing Gammaproteobacteria, *Nitrosococcus*, and *Nitrosomonas* (Supplementary Data 4). These genes could facilitate the transport of trehalose, as has been shown in *Mycobacterium tuberculosis* (Kalscheuer et al., 2010), which encodes an annotated trehalose transport system permease protein of 56.2% AAI (SugA) and 65.4% AAI (SugB) to the *Ca*. M. subdominans proteins. This transporter could perhaps provide *Ca*. M. subdominans with additional substrate options compared to *Ca.* M. parvicella. Trehalose synthesis and hydrolysis genes are present in both species, but transporter genes are absent in the *Ca.* M. parvicella MAGs, including *Ca.* M. parvicella RN1 (McIlroy et al., 2013). This compound could be used as a mechanism for managing environmental stress, including low temperatures, or serve as an energy storage compound (Elbein et al., 2003).

While *Ca.* M. parvicella prefers microaerobic conditions based on growth experiments (Rossetti et al., 2005), *Ca.* M. subdominans may be better adapted to oxic environments based on the genomic potential. Both *Ca.* M. subdominans and *Ca.* M. parvicella have the genomic potential to use nitrate or nitrite as a terminal electron acceptor under anoxic conditions as well as the microaerobic cytochrome *bd* oxidase and aerobic cytochrome *c aa*_3_ oxidase. However, *Ca.* M. subdominans encodes an additional *bc*_1_–*aa*_3_ type cytochrome super complex (*qcrABC*, *ctaCDEF*) in one syntenic region (Figure 8). Specifically, the QcrC component is a small di-heme cytochrome *c*, and has been found to be the only *c*-type cytochrome in most Actinobacteria (Kao et al., 2016), although both *Ca.* M. parvicella and *Ca.* M. subdominans also encode an additional mono-heme cytochrome *c* domain containing protein. This super complex is widespread in aerobic Actinobacteria, and is considered more bioenergetically efficient under aerobic conditions, enabling a direct link between menaquinol oxidation and dioxygen reduction (Kao et al., 2016; Davoudi et al., 2019). If these metabolisms are correct and active, *Ca.* M. subdominans may grow better in oxic conditions compared to the microaerophile *Ca.* M. parvicella.

### Ecophysiology of *Ca*. Microthrix and the Importance for Full-Scale WWTPs Performance

*Ca.* Microthrix has been known for decades by researchers and practitioners in the field, and *Ca.* M. parvicella has been considered the model organism for this genus. Our study has shown that there are only two abundant species present in municipal activated sludge plants across the world, and that an undescribed species, *Ca.* Microthrix subdominans, is usually present along with *Ca.* M. parvicella, but is generally less abundant. The abundance of both species may be strongly underestimated by using the commonly applied primer V4, compared to abundances obtained by the V1-V3 primers or by FISH, so the use of the V1-V3 primer set for future amplicon studies is highly recommended. Both species had very similar morphology and seem to impair wastewater settling properties, if abundant. *Ca.* Microthrix can also cause foaming due to its hydrophobic cell surface (de los Reyes, 2010). Foaming is, however, uncommon in Danish WWTPs, so we could not investigate whether the two species had different foaming properties.

The low level of variation in metabolic potential between the core genomes of *Ca.* M. parvicella and *Ca.* M. subdominans suggests that both species occupy a very similar niche in the WWTP ecosystem, yet unknown factors dictate the differences in their abundance and seasonal variation. This may result from the subtle differences in their genetic makeup or unknown conditions upstream of the WWTPs. Some detected genomic differences were related to the presence of a sugar transporter in *Ca.* M. subdominans, and the potential to use CO as an additional energy source, which may increase the chance for long-term survival during carbon starvation when organic carbon is not available (Cordero et al., 2019).

Wastewater treatment plants with nutrient removal promote the growth of *Ca.* Microthrix as indicated by our global survey. Such plants have dynamic oxic and anoxic conditions, which are selective for bacteria capable of storing carbon anaerobically, are microaerophilic, and can use nitrate and/or nitrite as electron acceptors, such as *Ca.* Microthrix. *Ca.* Microthrix is specialized in the uptake and storage of LCFAs, particularly under anoxic conditions, which allows them to successfully grow in the aerobic tanks with little external substrate present (Andreasen and Nielsen, 2000; Muller et al., 2014a; Roume et al., 2015). The storage compound present in *Ca.* M. parvicella has been suggested to be PHA in isolates and *in situ* based on Nile Blue A staining (Tandoi et al., 1998; Rossetti et al., 2002), while annotations of the two available genomes have yielded contradicting results (Muller et al., 2012; McIlroy et al., 2013). We verified that none of the *Ca.* Microthrix MAGs had the full set of genes for PHA storage, and that neither of the species possessed PHA *in situ*, as analyzed by Raman microspectroscopy. Instead we found the potential for TAG storage and observed an unspecified lipid-peak indicating storage of a lipid compound in both species, in agreement with our previous observations using isotope-labeled substrates (Nielsen et al., 2002). The results clearly indicate that both species of *Ca.* Microthrix are lipid accumulating bacteria, perhaps making them interesting targets for lipid recovery and subsequent biofuel conversion (Muller et al., 2014b).

*Ca.* M. parvicella is likely microaerophilic (Andreasen and Nielsen, 2000) as supported by our MAGs, while the additional cytochrome oxidase found in *Ca.* M. subdominans may potentially make this species less sensitive to higher oxygen levels. There is experimental evidence for the use of nitrate as an electron acceptor (Hesselsoe et al., 2005), although the annotations of the *nar* genes in the MAGs are ambiguous (McIlroy et al., 2013). In contrast, the *nirK* gene was more confidently annotated in all MAGs, so the details regarding *Ca.* Microthrix activity under anoxic conditions are still uncertain.

Poly-P granules have been commonly observed in *Ca.* Microthrix (e.g., Tandoi et al., 1998; Wang et al., 2014a,b), and their presence was confirmed *in situ* in both species. *Ca.* Microthrix could be a PAO, but the lack of active cycling of poly-P during anaerobic/aerobic phases, and the absence of a pit transporter across all the MAGs, indicates a less dynamic role in P removal. Instead, the poly-P reserves may be used for maintenance purposes as the granules are slowly depleted during longer starvation periods (Rossetti et al., 2002).

The global survey clearly demonstrated higher abundances of both species in countries with colder climates. This agrees with several studies (e.g., Knoop and Kunst, 1998; Sheik et al., 2016; Fan et al., 2017) and may be due to an increased availability of lipids to *Ca.* Microthrix compared to other species at low temperatures because of their hydrophobic surface as well as surface-associated lipases (Rossetti et al., 2005). Our study did not reveal obvious new ecophysiological traits that can be applied for the design of efficient control measures, but it provides a complete set of FISH probes and genomic information for future research into *in situ* gene expression and regulation, an important step toward understanding the ecology of *Ca.* Microthrix in WWTPs.

## Etymology

Description of *“Candidatus* Microthrix subdominans” sp. nov. (midas_s_2) “*Candidatus* Microthrix subdominans,” (sub.do’mi.nans. L. prep *sub* below; L. pres. part. *dominans* dominant; N.L. part. adj. *subdominans* indicating the abundance of this organism often below the dominant species *Ca.* Microthrix parvicella). This taxon was represented by the MAG Lyne_MB9. The complete protologue can be found in Supplementary Table 2.

## Data Availability Statement

Publicly available datasets were analyzed in this study. This data can be found here: sequencing data from Danish WWTPs is available at the Sequence Read Archive (https://www.ncbi.nlm.nih.gov/sra) under the project number PRJNA622675.

## Author Contributions

MN and PN devised the study and its main conceptual ideas. MN, CS, and MD performed data analysis with contributions from PN. FP and JP performed Raman measurements and data analysis. LT and ZK performed FISH analyses. MP and JP performed P-release lab experiments. The manuscript was drafted by MN and CS with contributions from FP, and revised by PN. All authors contributed to the article and approved the submitted version.

## Conflict of Interest

The authors declare that the research was conducted in the absence of any commercial or financial relationships that could be construed as a potential conflict of interest.

## Footnotes

1 https://www.midasfieldguide.org/guide/downloads

2 https://www.ncbi.nlm.nih.gov/sra

3 https://github.com/geronimp/enrichM
