# Supplementary material for: Low Global Diversity of Candidatus Microthrix, a Troublesome Filamentous Organism in Full-Scale WWTPs
Source: Front Microbiol. 2021 Jun 25;12:690251. doi: 10.3389/fmicb.2021.690251 (PMC8267870; doi:10.3389/fmicb.2021.690251)
Supplement: Supplementary file 1 [file Data_Sheet_1.pdf]

Supplementary Material:

Low Global Diversity of *Candidatus* Microthrix, a Troublesome Filamentous Organism in Full-Scale WWTPs.

Marta Nierychlo, Caitlin M. Singleton, Francesca Petriglieri, Lisette Thomsen, Jette F. Petersen, Miriam Peces, Zivile Kondrotaite, Morten S. Dueholm, Per H. Nielsen

Center for Microbial Communities, Department of Chemistry and Bioscience, Aalborg University, Aalborg, Denmark

Figure S1. The occurrence of the top 10 most abundant genera in Danish WWTPs with nutrient removal (page 2).

Figure S2. Composite FISH micrographs of *Ca*. Microthrix in full-scale activated sludge (page 2).

Figure S3. *Ca*. Microthrix read abundance comparison based on V1-V3 and V4 amplicon data at the (A) genus, and (B) species level (page 3).

Figure S4. Relative abundance of genera belonging to the family Microtrichaceae of all genera (in %) in activated sludge from global WWTPs (page 3).

Figure S5. Average nucleotide identity using BLASTN+ (ANIb) and alignment coverage of the genomes in *Ca.* M. parvicella and *Ca*. M. subdominans (page 4).

Table S1. Comparative genomics of the 10 MAGs and *Ca.* M. parvicella RN1 with 50% amino acid identity clustering at 80% alignment coverage (page 5).

Table S2. Protologue for *Candidatus* Microthrix subdominans (page 6).

Table S3. Average intracellular poly-P per µm filament in *Ca.* Microthrix and bulk ortho-P concentration during the dynamic poly-P uptake and release in anaerobic-aerobic cycles (page 9).

Data S1. MAG statistics for the MAGs in Figure 7. Including CDS regions, NCBI accession and taxonomy information (separate file).

Data S2. KOs and data used to create Figure 8 (separate file).

Data S3. KEGG modules identified with at least 80% completeness in the *Ca.* Microthrix MAGs and genomes (separate file).

Data S4. Top hits for the LpqY and SugABC proteins in *Ca*. M. subdominans using MAGE and the syntonome RefSeq analysis (separate file).

Note S1. Carbon monoxide oxidation (page 8).

Note S2. Evaluation of dynamic poly-P uptake and release in anaerobic- aerobic cycles in *Ca.* Microthrix (page 8).

References

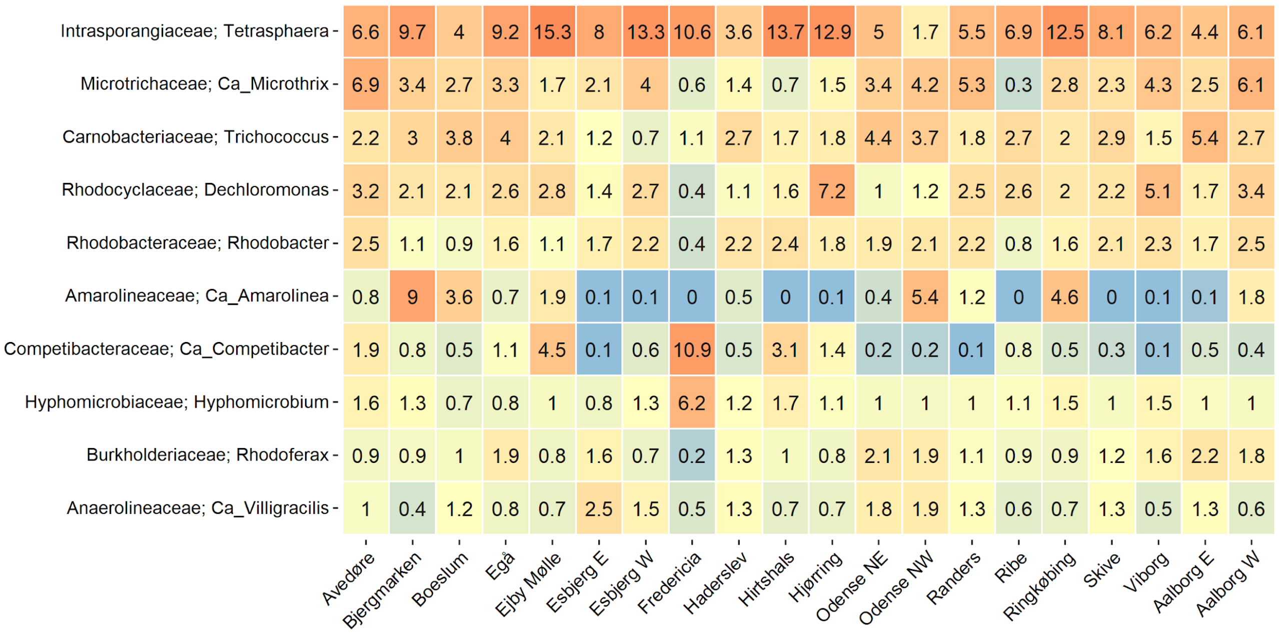

Figure S1. The occurrence of the top 10 most abundant genera in Danish WWTPs with nutrient removal. Family and genus names are shown. Data comes from long-term survey of microbial communities in the period 2006–2018 (Nierychlo et al., 2020a) containing 712 samples from 20 Danish nutrient removal WWTPs.

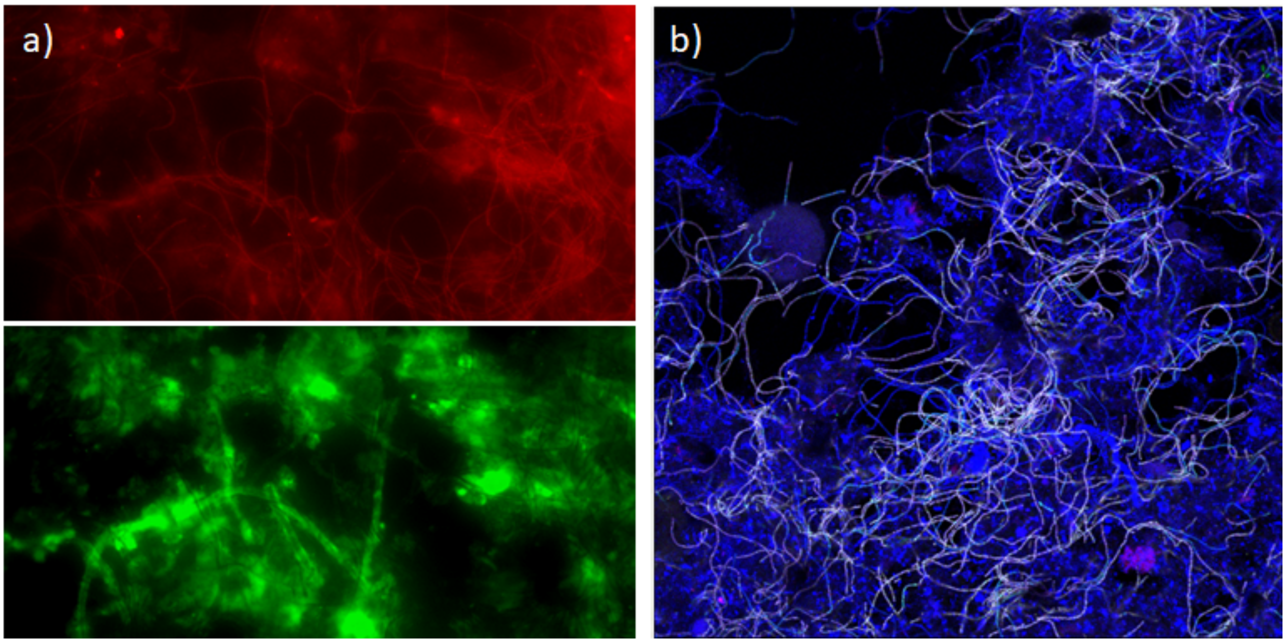

Figure S2. Composite FISH micrographs of *Ca*. Microthrix in full-scale activated sludge. (A) poor FISH signal from Mpa645 (Cy3, red, top panel) and corresponding EUBmix signal (6-FAM, green, bottom panel); (B) Overlap of species-specific probes Mpa60 (Cy3, magenta) and Mpa177 (6-FAM, cyan) targeting *Ca*. M. parvicella (appearing white). Other bacteria targeted with EUBmix (Cy5) appear blue. Activated sludge was sampled from Randers WWTP.

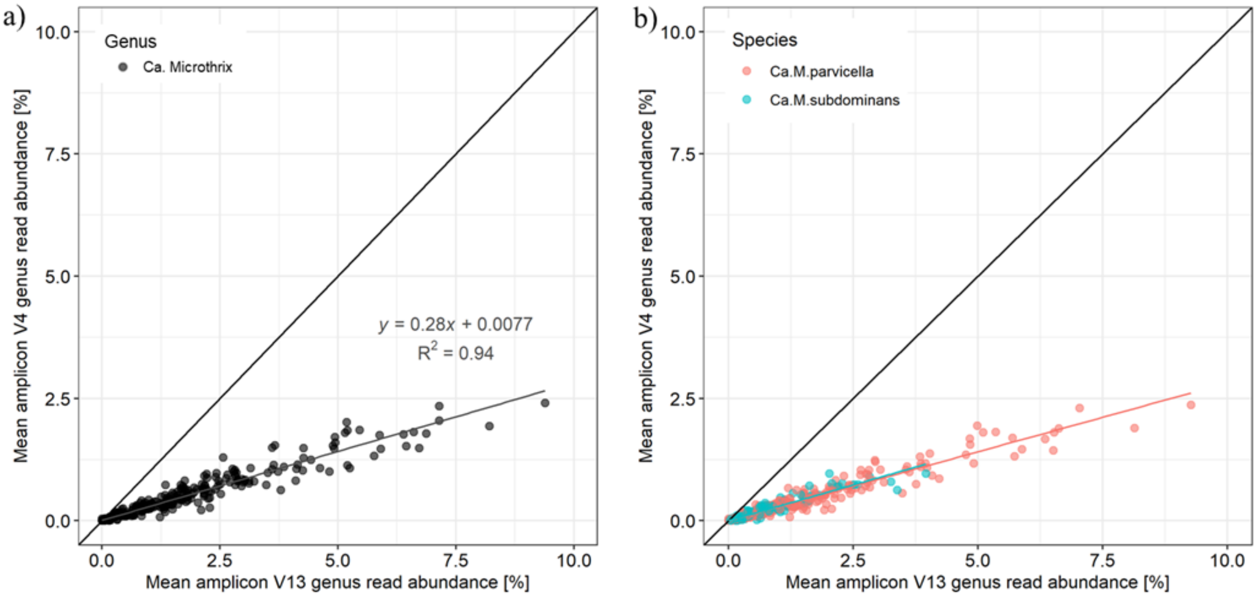

Figure S3. *Ca*. Microthrix read abundance comparison based on V1-V3 and V4 amplicon data at the (A) genus, and (B) species level. Data represents global MiDAS dataset (Dueholm et al., 2021) with total of 847 activated sludge samples from 438 plants with 4 different process designs (carbon removal plants, carbon removal and nitrification, carbon removal, nitrification and denitrification, and EBPR plants).

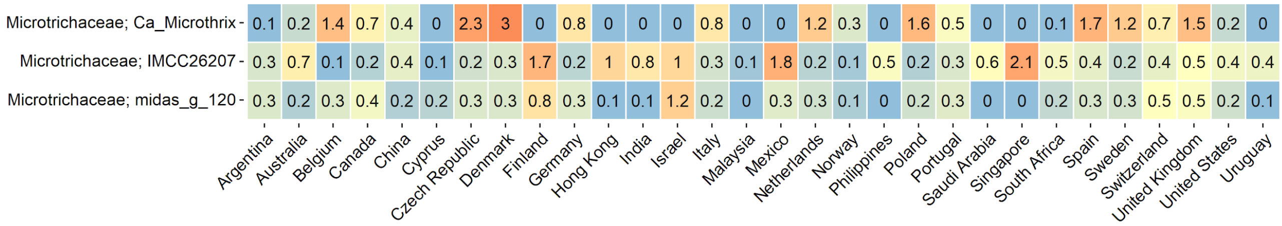

Figure S4. Relative abundance of genera belonging to the family Microtrichaceae of all genera (in %) in activated sludge from global WWTPs. Family and genus names are shown. Data represents global MiDAS dataset (Dueholm et al., 2021) with total of 847 activated sludge samples from 438 plants with 4 different process designs (carbon removal plants, carbon removal and nitrification, carbon removal, nitrification and denitrification, and EBPR plants).

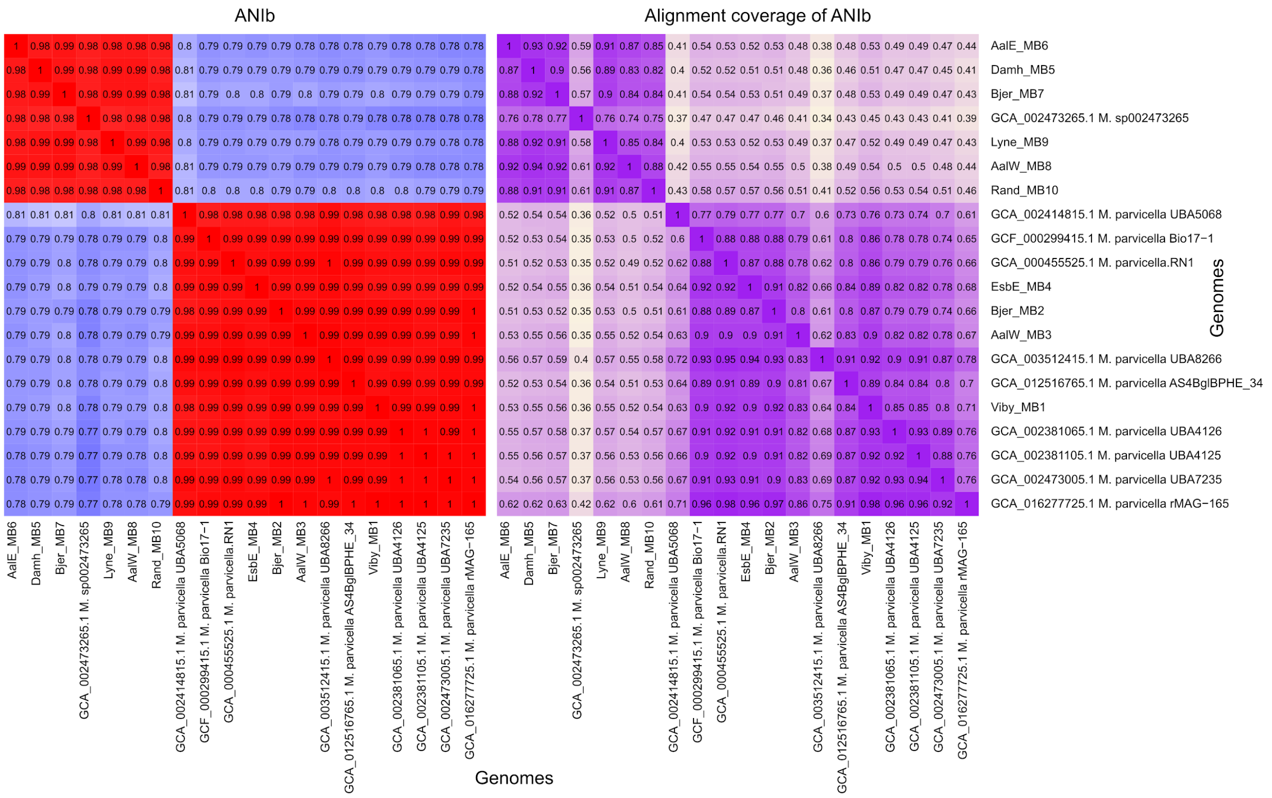

Figure S5. Average nucleotide identity using BLASTN+ (ANIb) and alignment coverage of the genomes in *Ca.* M. parvicella and *Ca*. M. subdominans. Genomes are ordered by their appearance in the genome tree.

Table S1. Comparative genomics of the 10 MAGs and *Ca.* M. parvicella RN1 with 50% amino acid identity clustering at 80% alignment coverage. Output was collected from Microbial Genome Analysis and Annotation Platform, Magnifying Genomes (MaGe).

**Group**	**Component**	**Families**	**Genes**	**Excluding**
Genus	Pan-genome	11765	47313	NA
Genus	Core-genome	1513	19428	NA
Genus	Variable-genome	10252	27885	NA
Ca. M. subdominans	Pan-genome	4974	9262	excluding *Ca*. M. parvicella clusters
Ca. M. subdominans	Core-genome	364	2281	excluding *Ca*. M. parvicella clusters
Ca. M. subdominans	Variable-genome	4610	6981	excluding *Ca*. M. parvicella clusters
Ca. M. parvicella	Pan-genome	3926	7077	excluding *Ca.* M. subdominans clusters
Ca. M. parvicella	Core-genome	380	1965	excluding *Ca.* M. subdominans clusters
Ca. M. parvicella	Variable-genome	3546	5112	excluding *Ca.* M. subdominans clusters

Table S2. Protologue for *Candidatus* Microthrix subdominans

**Taxonumber**	N/A
**Species name**	*Candidatus* Microthrix subdominans
**Genus name**	*Candidatus* Microthrix
**Specific epithet**	subdominans
**Type species of the genus**	*Candidatus* Microthrix parvicella
**ANI to type species of genus**	79% ANI with 53% alignment coverage to the genome of representative *Ca*. Microthrix parvicella Bio17.
**Taxonumber of the type species**	N/A
**Genus status**	Candidatus
**Species etymology**	sub.do'mi.nans. L. prep sub below; L. pres. part. dominans dominant; N.L. part. adj. subdominans indicating the abundance of this organism often below the dominant species *Ca.* Microthrix parvicella
**Species status**	sp. nov.
**Designation of the type MAG**	GCA_016719385.1
**MAG/SAG accession number**	GCA_016719385.1
**Genome status**	High-quality draft
**Genome size**	4214851
**GC mol %**	53.9
**CDS regions (determined using MAGE)**	4433
**Country of origin**	Denmark
**Region of origin**	Copenhagen
**Source of sample**	Full-scale biological nutrient removal wastewater treatment plant
**Geographical location**	Copenhagen
**Latitude**	55.695362 N
**Longitude**	12.612702 E
**Depth**	N/A

**Altitude**	N/A
**Temperature of the sample [In celcius degrees]**	Mesophilic
**pH of the sample**	N/A
**Relationship to oxygen**	Aerophile /microaerophile
**Energy metabolism**	Potentially utilizing lipids and CO, with a potential increased adaptability to aerobic conditions compared to *M. parvicella*
**Assembly**	1 sample
**Sequencing technology**	Oxford Nanopore and Illumina Hiseq X
**Binning software used**	Maxbin v2.2.7
**Assembly software used**	CANU v1.8
**Habitat**	Full-scale biological nutrient removal wastewater treatment plant
**Miscellaneous, extraordinary features relevant for the description**	Unbranched curled filaments with poly-P granules often present, 1 µm trichome width, and length often exceeding 90 µm.

Note S1 - Carbon monoxide oxidation

Ca. M. subdominans appears to encode several genes for a putative likely form II aerobic carbon monoxide dehydrogenase (CODH, *coxLMS*). The operon contains accessory genes *coxDEG*, and based on the active site of the CoxL (AYRGAGR) and the operon configuration of *coxSLM*, the enzyme encoded could belong to form II. A second orphaned copy of form II *coxL*, distantly related at 41% AAI to the first, is also present. Compared to model isolate organisms, the operon-based carbon monoxide dehydrogenase large subunit (CoxL) protein had an AAI of 40% to the CoxL of *Mycobacterium tuberculosis* H37Rv. The orphaned CoxL had 37.5% identity to *M. tuberculosis* H37Rv, and 43.71% AAI to the form I CoxL of the Chloroflexi *Thermomicrobium roseum*, a characterized CO oxidizer (Islam et al., 2019). Experimental work is required to confirm the capability of this form for CO oxidation. It is possible this enzyme could be involved in the oxidation of other compounds, as the active site is present in other molybdenum hydroxylases as well as the form II CODH (King and Weber, 2007). Carbon monoxide oxidation is suggested to be common in the mycobacteria, with slow growing populations oxidizing atmospheric levels of CO for energy during substrate-limiting conditions (King, 2003; King and Weber, 2007).

Note S2. Evaluation of dynamic poly-P uptake and release in anaerobic- aerobic cycles in *Ca.* Microthrix

Batch experiments were conducted on fresh activated sludge from Nakskov WWTP with many *Ca.* Microthrix to analyze total P and poly-P-content per cell of FISH-defined cells under dynamic anaerobic and aerobic conditions. Fresh activated sludge samples were aerated for 30 min to exhaust most intracellular carbon and refill poly-P reserves. After aeration, sludge was transferred to serum bottles and sealed with rubber stopper and aluminum cap. Pure nitrogen was used to flush the headspace in each bottle to ensure anaerobic conditions. The carbon source (oleic acid) was injected to the serum bottles after anaerobic conditions were ensured and were kept at room temperature (~22°C) with shaking for 3 h. Oleic acid was applied to provide a specific substrate for *Ca*. Microthrix (Andreasen and Nielsen, 1998) at a final concentration of 140 mg/L (0.5 mM). Oleic acid was heated at 60ºC and dissolved in detergent (0.02 mL Triton X100 in 100 mL oleic acid) prior addition to the serum bottles. Samples for ortho-P analysis and poly-P content were collected at the beginning of the experiment (0 h) and at the end of the experiment (3 h). The ortho-P released into the liquid phase was analysed in accordance with ISO 6878:2004 using the ammonium molybdate-based colorimetric method. Poly-P was quantified by Raman microspectroscopy in combination with FISH as described in the main manuscript (Section 2.5 and Section 2.7).

We observed that activated sludge with high abundance of *Ca.* Microthrix did release ortho-P when exposed to anaerobic conditions and oleic acid as carbon source (Table S3 and Figure below). However, none of the two *Ca.* Microthrix species released any significant amount of poly-P despite the high levels of intracellular poly-P before the anaerobic phase. Therefore, the ortho-P release was due to the activity of other PAOs in the activated sludge. This result is similar to previous findings, where *Ca.* Microthrix did not show any significant anaerobic release of poly-P when adding a mixture of acetate, glucose, and casamino acids in various EBPR sludges during aerobic-anaerobic cycling experiments (Petriglieri et al., 2021). These results show that although *Ca.* Microthrix species can take up large amounts of P, they are not canonical PAO with a dynamic release-uptake dynamic.

Table S3. Average intracellular poly-P per µm filament in *Ca.* Microthrix and bulk ortho-P concentration during the dynamic poly-P uptake and release in anaerobic-aerobic cycles.

	**Aerobic phase (0 h)**			**Anaerobic phase (3 h)**		
**Carbon source**	Bulk ortho-P [mgP/gSS]	*Ca.* M. parvicella Average poly-P per µm (*10^{-14} gP μm^{-1})	*Ca.* M. subdominans Average poly-P per µm (*10^{-14} gP μm^{-1})	Bulk ortho-P [mgP/gSS]	*Ca.* M. parvicella Average poly-P per µm (*10^{-14} gP μm^{-1})	*Ca.* M. subdominans Average poly-P per µm (*10^{-14} gP μm^{-1})
Oleic acid	0.81 ± 0.13	1.58 ± 0.03	1.57 ± 0.01	3.03 ± 0.34	1.37 ± 0.03	1.40 ± 0.03
Blank	0.81 ± 0.13	-	-	1.80 ± 0.01	-	-

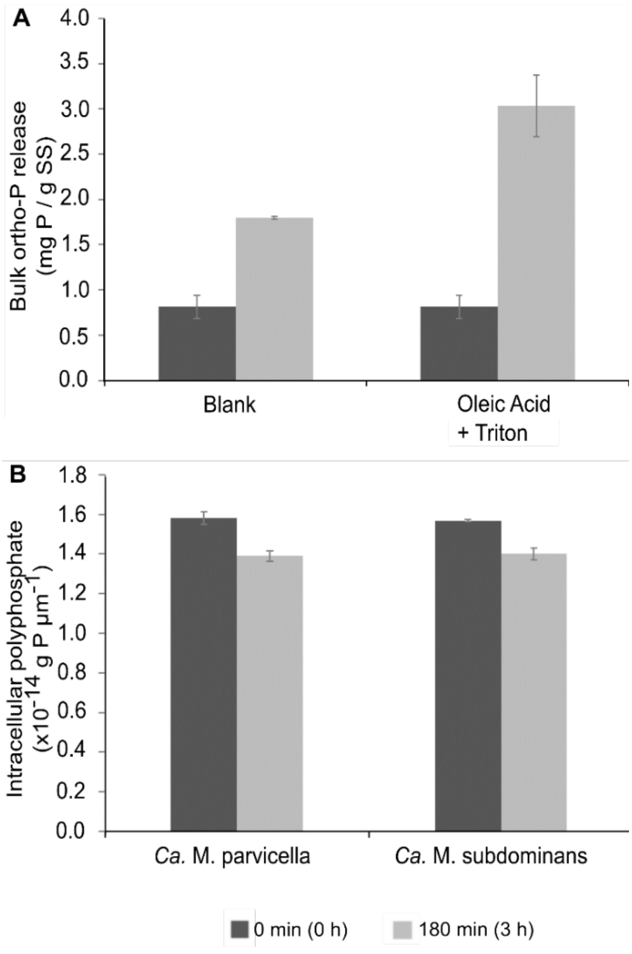

Figure. A) Bulk ortho-P concentration during anaerobic P-release experiments with activated sludge from Nakskov WWTP. B) Average intracellular poly-P per µm filament in the two most dominant *Ca.* Microthrix species measured as an average of around 250 randomly selected microbial cells by Raman microspectroscopy in initial 0 h samples and after 3 h anaerobic P-release with oleic acid + triton as substrate. Blank without added substrate is also shown for ortho-P release.

References

Dueholm, M. S., Nierychlo, M., Andersen, K. S., Rudkjøbing, V., Knudsen, S., the MiDAS Global Consortium, et al. (2021). MiDAS 4 - a global WWTP ecosystem-specific full-length 16S rRNA gene catalogue and taxonomy for studies of bacterial communities. *BioRxiv*.

Islam, Z. F., Cordero, P. R. F., Feng, J., Chen, Y.-J., Bay, S. K., Jirapanjawat, T., et al. (2019). Two Chloroflexi classes independently evolved the ability to persist on atmospheric hydrogen and carbon monoxide. *ISME J.* 13, 1801–1813. doi:10.1038/s41396-019-0393-0.

King, G. M. (2003). Uptake of carbon monoxide and hydrogen at environmentally relevant concentrations by Mycobacteria†. *Appl. Environ. Microbiol.* 69, 7266–7272. doi:10.1128/AEM.69.12.7266-7272.2003.

King, G. M., and Weber, C. F. (2007). Distribution, diversity and ecology of aerobic CO-oxidizing bacteria. *Nat. Rev. Microbiol.* 5, 107–118. doi:10.1038/nrmicro1595.

Nierychlo, M., Andersen, K. S., Xu, Y., Green, N., Jiang, C., Albertsen, M., et al. (2020a). MiDAS 3: An ecosystem-specific reference database, taxonomy and knowledge platform for activated sludge and anaerobic digesters reveals species-level microbiome composition of activated sludge. *Water Res.* 182, 115955. doi:10.1016/j.watres.2020.115955.

Petriglieri, F., Petersen, J. F., Peces, M., Nierychlo, M., Hansen, K., Baastrand, C. E., et al. (2021). Quantification of biologically and chemically bound phosphorus in activated sludge from full-scale plants with biological P-removal. *BioRxiv*. doi:10.1101/2021.01.04.425262.
